# Resveratrol-Loaded Nanoemulsions: In Vitro Activity on Human T24 Bladder Cancer Cells

**DOI:** 10.3390/nano11061569

**Published:** 2021-06-15

**Authors:** Federica Rinaldi, Linda Maurizi, Jacopo Forte, Massimiliano Marazzato, Patrizia Nadia Hanieh, Antonietta Lucia Conte, Maria Grazia Ammendolia, Carlotta Marianecci, Maria Carafa, Catia Longhi

**Affiliations:** 1 Dipartimento di Chimica e Tecnologie del Farmaco, Sapienza Università di Roma, P.le A. Moro 5, 00185 Roma, Italy; federica.rinaldi@uniroma1.it (F.R.); jacopo.forte@uniroma1.it (J.F.); patrizianadia.hanieh@uniroma1.it (P.N.H.); maria.carafa@uniroma1.it (M.C.); 2 Dipartimento di Sanità Pubblica e Malattie Infettive, Sapienza Università di Roma, P.le A. Moro 5, 00185 Roma, Italy; linda.maurizi@uniroma1.it (L.M.); massimiliano.marazzato@uniroma1.it (M.M.); antoniettalucia.conte@uniroma1.it (A.L.C.); catia.longhi@uniroma1.it (C.L.); 3 Centro Nazionale Tecnologie Innovative in Sanità Pubblica, Istituto Superiore di Sanità, Viale Regina Elena, 299, 00162 Rome, Italy

**Keywords:** resveratrol, bladder cells, essential oils, nanoformulations, nanoemulsions, bioactive compound, cytotoxicity activity

## Abstract

The chemopreventive potential of Resveratrol (RV) against bladder cancer and its mechanism of action have been widely demonstrated. The physicochemical properties of RV, particularly its high reactivity and low solubility in aqueous phase, have been limiting factors for its bioavailability and in vivo efficacy. In order to overcome these limitations, its inclusion in drug delivery systems needs to be taken into account. In particular, oil-in-water (O/W) nanoemulsions (NEs) have been considered ideal candidates for RV encapsulation. Since surfactant and oil composition can strongly influence NE features and their application field, a ternary phase diagram was constructed and evaluated to select a suitable surfactant/oil/water ratio. The selected sample was deeply characterized in terms of physical chemical features, stability, release capability and cytotoxic activity. Results showed a significant decrease in cell viability after the incubation of bladder T24 cancer cells with RV-loaded NEs, compared to free RV. The selected NE formulation was able to preserve and improve RV cytotoxic activity by a more rapid drug uptake into the cells. O/W NEs represent an effective approach to improve RV bioavailability.

## 1. Introduction

Among phytochemicals, phytoestrogens have been reported to contain several bioactive molecules. These compounds can be classified into four main groups, such as isoflavonoids, flavonoids, lignans and stilbenes. Resveratrol, derived from red grapes, berries, and peanuts, belonging to the stilbene group, has a 14-carbon skeleton consisting of two aromatic rings with hydroxyl groups in position 3, 5, and 4′, joined by a double styrene bond. The double bond is responsible for the isometric *cis*- and *trans*-forms of resveratrol. The *trans*-isomer appears to be the more predominant and stable natural form [1,2].

In the last few years, RV has been subjected to considerable attention for its pleiotropic activities [3]. Clinical studies have demonstrated several health-promoting activities of RV, such as antioxidizing and anti-atherosclerotic effects, inhibition of platelet aggregation, beneficial effects on the cardiovascular system, reducing lipid peroxidation, improving vasodilatation, and lowering blood pressure [4].

RV is currently being evaluated as a potential cancer chemoprevention agent against several kinds of tumors such as leukemia, prostate, breast, colon and bladder cancers [5,6,7,8].

A preliminary study investigated the chemopreventive potential of RV against bladder cancer and its mechanism of action [9]. As reported by the authors, RV was able to induce apoptosis through the modulation of Bcl-2 family proteins and activation of caspase 9 and caspase 3 followed by poly (ADP-ribose) polymerase degradation. Treatment with RV led to G1 phase cell cycle arrest in bladder T24 cells by activation of p21 and downregulation of cyclin D1, cyclin-dependent kinase 4, and phosphorylated Rb. RV also inhibited the phosphorylation of Akt, whereas the phosphorylation of p38 MAPK was enhanced. In addition, RV treatment decreased the expression of vascular endothelial growth factor and fibroblast growth factor-2, which might contribute to the inhibition of tumor growth on the bladder cancer xenograft model [9].

The physicochemical properties of RV, particularly its high reactivity and low solubility in aqueous and lipid phases, have been limiting factors for its bioavailability and the efficacies of the desired health-beneficial effects. In a biological system, RV is rapidly and extensively metabolized, probably due to its low water solubility, which reduces the dissolution rate’s limited cell absorption, thus reducing its oral bioavailability [4].

To overcome RV bioavailability limitations, different delivery strategies have been described in the literature [10,11,12]. Among proposed formulations, oil-in-water (O/W) nanoemulsions (NEs) have been considered ideal candidates for the encapsulation of RV because of their easy fabrication by high-energy processes and the possibility of using natural ingredients with low concentrations of emulsifiers [13,14,15]. Immobilization of active compounds in the lipid matrix of the NEs efficiently contributes to improving the dispersibility of the bioactive compounds in aqueous solutions, minimizing the tendency to separate the different phases (aqueous and lipid) and to improve absorption and bioavailability [16]. Nowadays, nanoemulsions are considered as an attractive drug delivery system with respect to the others, for many reasons. Two major distinguishing advantages of nanoemulsions are their stability and easy penetration in the cellular membrane. Moreover, these nanocarriers offer high solubilizing potential for lipophilic and hydrophilic drugs with consequent high values of entrapment efficacy. Nanoemulsions are composed of oil, surfactant and a water phase [17]. The choice of oil and surfactant pair is of crucial importance for the design and preparation of the desired nanoemulsion and, at the same time, for preparing functional NEs. In particular, oil properties can have a synergic effect with the entrapped drug in order to enhance the activity of the overall system, and in which the surfactant concentration should be kept as low as possible to balance between drug permeation and their potential pharmaceutical activity (e.g., oleic acid in Tween 80 surfactant could contribute to an antioxidant activity) and no or less toxic effects [18]. Another nanoemulsion advantage is that preparation methods are generally simple and low cost.

RV-loaded NEs (NR) are characterized by the droplet size of the dispersed phase, ranging from 20 to 200 nm, and they are obtained by a high energy method of preparation (e.g., sonication). Moreover, the NEs maintain the same size range when non-diluted and diluted [19]. Typically, NEs contain oil, water, and an emulsifier, and may offer higher solubilization and improved bioavailability of poorly soluble active substances, and also a system to preserve the volatile compounds of essential oils [20]. The knowledge gained from this research should improve our understanding of the factors that influence the formation, stability, and utilization of nanoemulsions as delivery systems for bioactive compounds. For this purpose, construction of the pseudoternary phase diagram is fundamental in order to evaluate and select the best NE formulation, in terms of composition and physical–chemical features.

In particular, the aim of this study was to design and characterize nanoemulsions, consisting of non-ionic surfactant and Neem seed oil, as delivery systems to encapsulate RV, and study the interaction of these delivery systems with in vitro model human T24 bladder tumor cells.

NR were deeply characterized in terms of size and ζ-potential, stability over time, morphology, microviscosity and polarity. Moreover, RV release experiments in culture media were carried out and the effect of RV-free or loaded in NEs on the T24 cell line was evaluated by a cytotoxicity assay. Trans stilbene (TS)-loaded NEs (NT) were used as a blank, because they show the same physical–chemical characteristics in comparison to NR, but not the same therapeutic efficacy.

## 2. Materials and Methods

### 2.1. Materials

Tween 20 (Tw20), 3,5,4’-trihydroxy-*trans*-stilbene (RV), *trans*-1,2-diphenylethylene (TS), human serum (HS), Hepes salt (*N*-(2-hydroxyethyl) piperazine-*N*-(2-ethanesulphonic acid)), pyrene, Nile Red and Roswell Park Memorial Institute (RPMI) 1640 medium were Sigma-Aldrich products (Sigma-Aldrich, Milan, Italy). Neem oil was purchased from Neem Italia (Moniga del Garda (BS), Italy) and characterized by an ECOCERT certificate (Biocert Italia IT013BC041—ICEA 264BC001). All other products and reagents were of analytical grade.

### 2.2. Pseudoternary Phase Diagram Construction and Nanoemulsion Preparation

The pseudoternary phase diagram of Neem seed oil NEs was developed. The mixtures were prepared by combining appropriate amounts of surfactant, oil phase, and aqueous phase (Hepes buffer, pH 7.4) in different weight ratios (Table S1), in a test tube, and vortexed vigorously for 5 min to ensure thorough mixing. Visual inspection was made after each sample preparation. The NE formulations were prepared using Tween-20 and Neem oil in 5 mL of Hepes buffer (10^−2^ M, pH 7.4), in an oil/surfactant ratio of 1:1. The mixture was vortexed for about 5 min to allow the micro-emulsion to form, and then, the obtained microscale droplets were sonicated for 20 min at 50 °C, using a tapered microtip operating at 20 kHz at an amplitude of 18% (Vibracell-VCX 400, Sonics, Taunton, MA, USA), to obtain the NEs. At this stage, all formulations can be sterilized by using cellulose filters (0.22 µm) in accordance with Ph. Eur. (Table S1).

### 2.3. Preparation of Selected Nanoemulsions

The sample NT was selected by the homogenous phase (blue zone) of ternary diagram and prepared as reported in a previous work [21].

The active compound, RV/TS, or lipophilic probe (Nile Red) was previously solubilized in the Neem seed oil (Table 1) and the surfactant was added to the oil/active compound at a weight ratio of 2:1 (*w/w*) to obtain NR, NT and Nile Red samples. Hepes buffer (10^−2^ M, pH 7.4) was joined as aqueous phase and the obtained mixture was vortexed for about 5 min, allowing the formation of a homogeneous dispersion.

The empty, RV- or Nile Red-loaded samples were sonicated for 20 min at 50 °C using a tapered microtip operating at 20 kHz at an amplitude of 18% (Vibracell-VCX 400, Sonics, Taunton, MA, USA) to obtain, respectively, N, NR or Nile Red samples. The sample containing TS was sonicated at room temperature for 5 min to obtain NT sample. The final concentration of RV and TS entrapped into NEs was measured by using UV spectrophotometer (Lambda 25, PerkinElmer, Waltham, MA, USA) at 270 nm, equipped by 1.0 cm path-length quartz cells. In particular, the active compound concentration was calculated by using the calibration curves previously determined.

### 2.4. Dynamic Light Scattering Measurements

Hydrodynamic diameter and polydispersity index (PDI) of NE formulations were analyzed at 25 °C by using a ZetaSizerNano 90 S (Malvern Instruments Ltd., Worcestershire, United Kingdom), equipped with a 5 mW HeNe laser (wavelength λ = 632.8 nm) and a digital logarithmic correlator. The normalized intensity autocorrelation functions were detected with an angle of 90° and analyzed by a Contin algorithm to obtain the decay time of the electric field autocorrelation functions [22]. By using the decay time, it is possible to obtain the distribution of the diffusion coefficient D of the particles and with the Stokes–Einstein relationship (RH = KBT/6πηD, where KBT is the thermal energy and η the solvent viscosity), the effective hydrodynamic radius RH is determined. The values of the radii reported in this paper correspond to the intensity weighted average [23].

The absolute ζ-potential value of nanoemulsions was measured by electrophoretic mobility using the Smoluchowski equation ζ = uη/є, where η and є are the viscosity and the permittivity of the solvent phase [24].

### 2.5. Stability Studies

In order to evaluate NE colloidal stability over time, all samples were stored up to 30 days at two different temperatures (room temperature and 4 °C). The samples were analyzed following the variation of hydrodynamic diameter and ζ-potential at definite time intervals (1, 7, 15 and 30 days).

Furthermore, the effect of biological media on NE stability was studied. In particular, mixtures of NEs and 45% of RPMI 1640 medium (Sigma, St. Louis, MO, USA) were prepared and incubated at 37 °C. Analyses were performed at different time intervals up to 48 h by DLS monitoring the variations of particle size and ζ-potential. The characteristic RT UV spectrum was evaluated to obtain qualitative and quantitative information on entrapment variation and hypothetical degradation phenomena. The study was carried out up to 30 days at room temperature and 4 °C.

### 2.6. Fluorometric Measurements

Oil droplet features were investigated by using fluorescent probes. In particular, pyrene-loaded NEs were prepared by adding the probe (4 mM) to the oil phase, using the same preparation method described above.

This fluorescent probe shows a spectrum characterized by five emission peaks (I_1_–I_5_) as monomers, while its excimer exhibits only one peak (I_E_), and their fluorescence intensities depend on the lateral distribution and mobility of the probe into the oil phase. In particular, the ratio of the I_1_/I_3_, corresponding to the first and third vibration bands in the pyrene spectrum, is related to the polarity of the probe environment. In fact, according to the viscosity of the probe environment, pyrene can form the intramolecular excimer [25] and it can be calculated by the ratio between I_E_ and I_3_. The fluorescence signals emitted by pyrene-loaded NEs were scanned (λ = 350–550 nm) by using a luminescence spectrometer (LS5013, PerkinElmer, Waltham, MA, USA).

### 2.7. Release Studies in RPMI 1640 Medium

In vitro release experiments were carried out using the dialysis technique. In particular, NEs and 10% RPMI 1640 medium were placed inside a dialysis bag (cellulose membrane, molecular weight cut-off 8000 and 5.5 cm^2^ diffusing area) and immersed in a solution (external medium) composed of Hepes buffer and ethanol (50:50). The releasing system was maintained under magnetic stirring at 37 °C. At pre-determined time intervals (each hour, from 0 to 8 h, and after 24 h), aliquots from the external medium were taken out and analyzed with a spectrometer (Lambda 25, PerkinElmer, Waltham, MA, USA) in order to evaluate the concentration of RV or TS released. The experiments were carried out in triplicate for both samples.

### 2.8. NE Transmission Electron Microscopy

Diluted NE formulations were layered onto carbon-coated copper grids and stained with 2% (*v/v*) phosphotungstic acid (PTA), adjusted to pH 7.0. Imaging was performed at an acceleration voltage of 80 kV with an FEI 280 S transmission electron microscope (FEI, Eindhoven, The Netherlands). Adobe Photoshop software was used to optimize image editing.

### 2.9. Preparation of Polyphenols Solutions

RV was dissolved in 100 µL of 95% ethanol (EtOH) and stored at −20 °C. TS was prepared by solubilizing 7.2 mg in 1 mL of 95% EtOH. For all experiments, the final concentrations of polyphenols were prepared by diluting the stock in culture medium.

### 2.10. Cell Culture

The T24 human bladder cancer cell line was obtained from the American Type Culture Collection (Manassas, VA, USA). The cells were cultured in RPMI 1640 medium supplemented with 10% heat-inactivated fetal bovine serum (FBS; SAFC Biosciences Inc., Lenexa, KS, USA), 100 IU/L penicillin and 100 mg/L streptomycin. Cultures were maintained in a humidified atmosphere containing 5% CO_2_ at 37 °C.

### 2.11. NE Fluorescence Assay

To visualize NE uptake by T24 cells, NEs loaded with Nile Red dye (NEs-NR) were prepared [26]. T24 cells seeded on 8-well chamber-slides (Falcon) for 24 h at 37 °C were exposed to either Nile Red NEs or free Nile Red, prepared as 1 mg/mL stock solution in acetone and used at a final concentration of 100 ng/mL. After 7 or 24 h incubation, cells were washed with phosphate-buffered saline solution (PBS), pH 7.4 and fixed in methanol/acetone (1:1) for 5 min at −20 °C. After three washes with PBS, slides were mounted with 0.1% (*w/v*) p-phenylenediamine in 10% (*v/v*) PBS, 90% (*v/v*) glycerol, pH 8.0 and observed by fluorescence microscopy using a Leica DM4000 (Leica Microsystem, Wetzlar, Germany) fluorescence microscope, equipped with an FX 340 digital camera.

### 2.12. Effect of Free or NE Loaded RV on T24 Cells by MTT Assay

T24 cells (concentration of 1 × 10^4^/well) were seeded in 96-well plates and cultured at 37 °C with 5% CO_2_ for 24 h. Free RV and TS or NR/NT at different concentrations were then added to the monolayers. After 24 and 48 h, the supernatant was discarded and 100 μL of 0.5 mg/mL of 3-(4,5-dimethylthiazol-2-yl)-2,5-diphenyltetrazolium bromide (MTT) reagent was added for an additional 4 h. Afterwards, the supernatant was sucked out, and cells were lysed in 200 μL DMSO for 10 min at room temperature. Optical density (O.D.) at 568 nm was measured using a microplate reader (PerkinElmer, Boston, MA, USA).

### 2.13. Effect of RV/TS or NR/NS on T24 Cells by Trypan Blue Assay

T24 cells (2 × 10^5^/mL) were seeded in 12-well plates and cultured at 37 °C and 5% CO_2_ for 24 h; then, 25 and 50 µM of RV and TS, alone or loaded in NEs, were added to the cells. After culturing for 24 and 48 h, the cells were trypsinized and 20 µL of each sample were mixed with 60 µL of Trypan Blue and cells were counted by a hemocytometer.

### 2.14. Reactive Oxygen Species (ROS) Production

Intracellular ROS production was detected by use of the oxidant-sensitive fluorescent probe 2′, 7′ dichlorodihydrofluorescein diacetate (H_2_DCF-DA), which is freely permeable to the cell membrane [27]. Experiments were performed in 96-well plates seeded with T24 cells. After 7 and 24 h treatment with RV/TS alone or loaded in NEs, cells were washed with PBS and then incubated with 10 µM H_2_DCF-DA for 30 min at 37 °C. As a positive control, staurosporine 1 μM was used. After incubation, cells were washed with PBS and fluorescence intensity was measured in a microplate reader (FLUOstar, BMG France). The excitation/emission wavelength was set at 495/520 nm. The result of the assay was expressed as percentage of the relative fluorescence with respect to the untreated control cells.

### 2.15. Statistical Analysis

Each experiment was performed in triplicate, and all values were reported as mean ± standard deviation (SD). The Kruskal–Wallis test followed by Dunn’s post hoc pairwise test was used to assess statistical significance, while the Wilcoxon signed-rank test was employed to compare condition over consecutive time points. Where necessary, the *p* values were corrected with the Benjamini–Hockberg procedure in order to account for multiple comparisons. A *p* value ≤ 0.05 was considered statistically significant.

## 3. Results and Discussion

### 3.1. NE Design and Characterization

**Figure 1:**
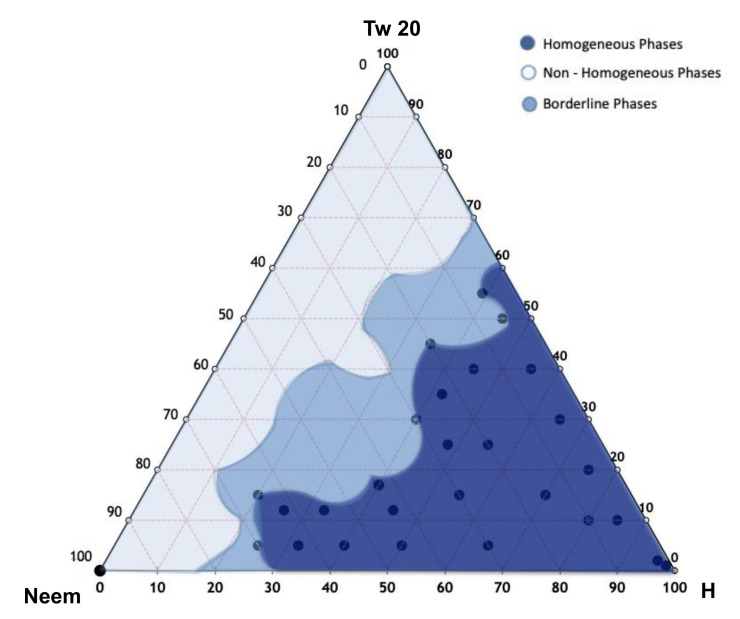
Ternary phase diagrams between Neem oil, Tween 20, and Hepes buffer. The resulting phases observed were the homogeneous phase (dark blue area), the non-homogenous phase (light blue area) and the borderline phase region (blue area). NE tested compositions have been chosen in the dark blue region.

The pseudoternary phase diagram of Neem oil NEs was developed, and different homogeneous phase regions were identified, in order to select the appropriate NEs in terms of hydrodynamic diameter, ζ-potential, and PDI. In Figure 1, the ternary phase diagrams of Neem oil with Tween-20 and Hepes buffer are shown. A homogeneous phase, according to a visual inspection, can be obtained by mixing different amounts of Neem oil, Tween-20, and Hepes buffer. Ternary phase diagram construction is the best way to observe the formation of homogeneous dispersion by mixing these three components. This study is performed to select the optimized amounts of surfactant, oil, and Hepes buffer in the development of Neem NEs. Figure 1 highlights the presence of three different regions corresponding to homogeneous dispersions (dark blue region) and non-homogeneous dispersions, characterized by phase separation phenomena (light blue zone). Inside the light blue zone, only for some formulations, the sonication leads to the formation of monophasic dispersions (blue zone) [28,29,30]. To optimize the monophasic emulsions as a suitable drug delivery system (NEs), all samples in the dark blue region were sonicated for 20 min at 50 °C. The best formulation in terms of hydrodynamic diameter, ζ-potential, and PDI was selected and observed by TEM.

### 3.2. Characterization Studies: NE Physical–Chemical Features

**Figure 2:**
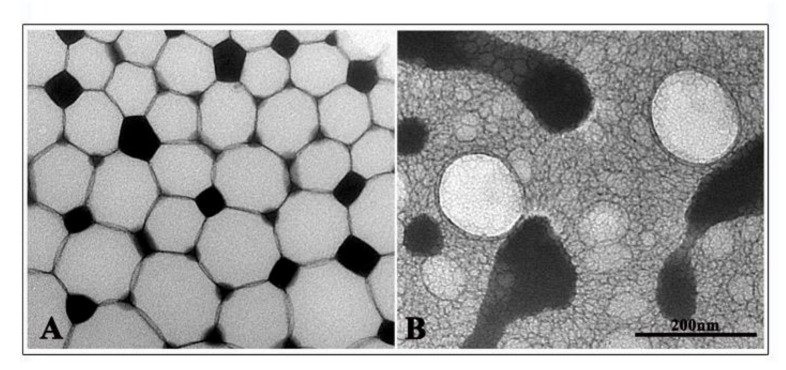
Transmission electron microscopy micrograph of NR (panel **A**) and NT (panel **B**).

The obtained NEs were characterized in terms of size, ζ-Potential, PDI and RV/TS entrapment efficiency (Table 2). Dynamic light scattering analyses indicated a comparable increase in RV- and TS-loaded NEs’ dimensions in comparison with the empty ones (from 38.8 nm to 137.8/156.2 nm), because even the amount of RV and TS entrapped is in the same range value. PDI values were not affected by the RV and TS internalization and remain for all samples around 0.2, confirming the monodispersity of all NE formulations. TEM observations showed a transition from the micro- to nanoemulsion following sonication. Empty NEs appeared to be formed from a microemulsion sample consisting of microscale vesicles and structured as spherical droplets with size according to those revealed by DLS (Figure S1). These NEs, when loaded with RV, appeared to maintain a spherical shape, with a regular arrangement, and partially deformed because tightly attached. TS NEs also appeared spherical in shape and enclosed in a matrix of droplets with smaller sizes. NE sizes recorded by TEM were comparable with those observed by DLS analyses for both samples (Figure 2).

In order to characterize the nature of the lipophilic droplet, microviscosity and polarity have been evaluated. Pyrene-loaded NEs were prepared following the procedure previously described and the fluorescence spectrum allowed the calculation of the values of polarity and microviscosity reported in Table 2. Microviscosity values are quite similar for all NEs, but polarity values increase with RV/TS inclusion. It is likely that the hydroxyl groups of RV/TS inside the oil droplet affect its polarity and not its microviscosity, because of the similar drug chemical structures, low molecular weight and reduced steric hindrance of the two molecules as well as inclusion at comparable drug-loaded concentration.

Knowledge of oil droplet characteristics is a fundamental aspect to investigate in order to better understand and predict the in vivo interaction of NEs with biological milieu such as membranes, plasma proteins and cells.

### 3.3. NE Stability

**Figure 3:**
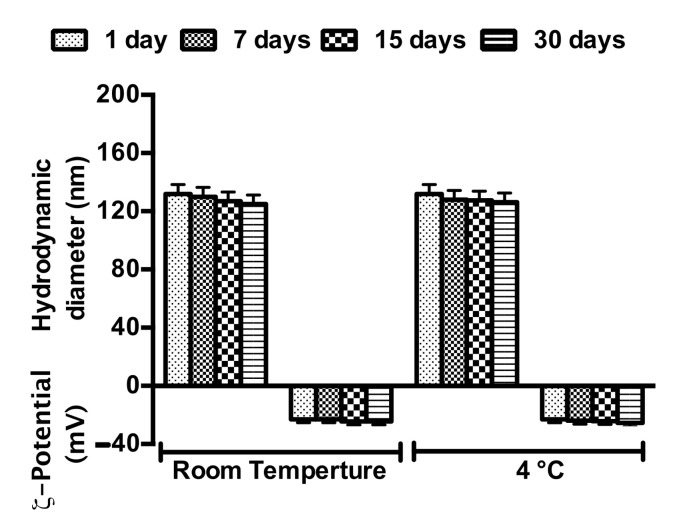
Stability studies of NR in terms of hydrodynamic diameter and ζ-potential up to 30 days at two different storage temperatures.

**Figure 4:**
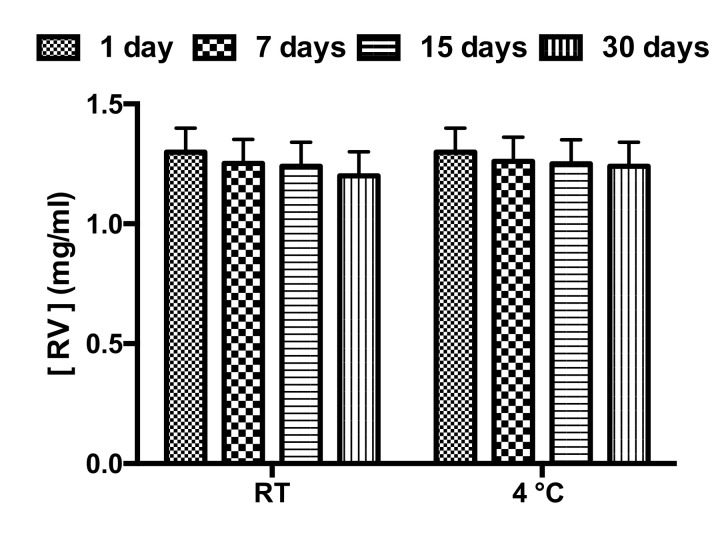
RV-loaded NEs: stability over time at two different temperatures—room temperature (RT) and 4 °C.

The physical stability of NEs must be taken into account because the evaluation of NE stability or NE chemical–physical features (Table 2) is fundamental to determine their application field or administration route. Stability studies of NR and NT were performed according to the method previously described. NR (Figure 3) and NT (Figure S2) appeared to be stable for at least 30 days when stored at both experimental temperatures (25 °C and 4 °C). The biological stability of NT and NR samples was also evaluated in RPMI 1640 medium. Both samples were stable at the temperature of 37 °C until up to 48 h (Figure S3). In order to evaluate any degradation or release phenomena affecting RV after inclusion in NEs, RV spectra were recorded immediately after sample preparation up to 30 days. RV amount did not vary in the analyzed time interval at room temperature and at 4 °C, no degradation occurred (Figure 4). One of the main goals of such delivery system is to overcome the drawbacks of RV, enhancing its stability by inclusion in oil droplets dispersed in an aqueous phase: O/W NEs. The enhanced stability and increased water solubility (by NEs inclusion) can ameliorate RV bioavailability and hence, its efficacy. The similar oil droplet characteristics of the NT and NR samples have also been confirmed by the same stability trend over time (NT Figure S2).

### 3.4. RV Release Studies in RPMI 1640 Medium

**Figure 5:**
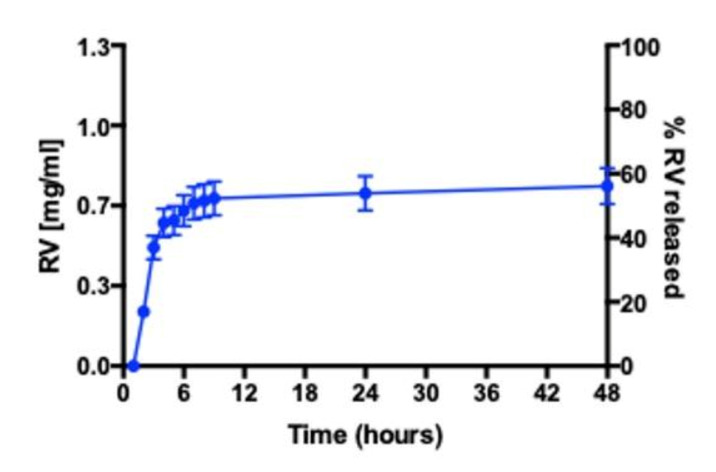
RV release profile by NR in RPMI 1640 medium.

The total amount of RV (Figure 5) and TS released by NEs in RPMI 1640 medium is around 50%. In particular, the RV released reaches the maximum amount within 8 h (Figure 5). Moreover, it is possible to highlight that samples are characterized by similar release profiles, probably due to having the same physical–chemical characteristics of RV/TS. The release studies confirmed the ability of NEs to release RV in order to obtain the desired effect in in vitro cell studies. After 48 h, NE integrity was evaluated in the donor compartment by DLS analyses, confirming no sample degradation during the experiment in RPMI 1640 medium (data not shown).

### 3.5. Cell Internalization of NE Loaded with Nile Red by Fluorescence Assay

**Figure 6:**
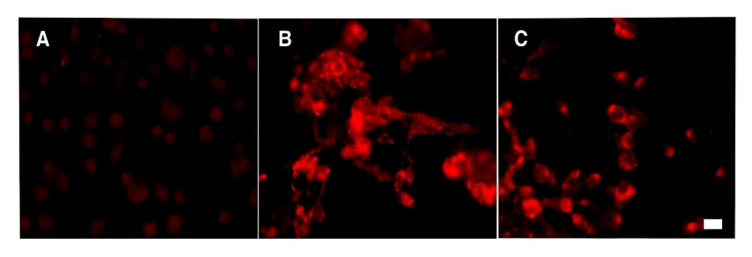
Nile Red NE visualization after 24 h cell treatment. Panel (**A**) control cells treated with free Nile Red. Panel (**B**) T24 cells treated with Nile Red 30 nm NEs. Panel (**C**) T24 cells treated with Nile Red 140 nm NEs. Bar, 10 μm.

In order to evaluate the influence of size on cellular uptake, NEs (30 nm or 140 nm) loaded with Nile Red were put in contact with semi-confluent T24 cell monolayers for 7 or 24 h and analyzed by fluorescence microscopy. As previously demonstrated for Hep2 monolayers [26], selected nanoemulsion formulations loaded with Nile Red were internalized in the T24 cell line. The results obtained showed a strong fluorescent intracellular signal in cells exposed to NEs, as a punctuated pattern increasing with the time of incubation. Compared to 30 nm NEs, 140 nm NEs showed a lower cellular uptake, as expected [31] (Figure 6).

### 3.6. Effect of Free RV or Loaded in NEs towards T24 Bladder Cell Monolayers

**Figure 7:**
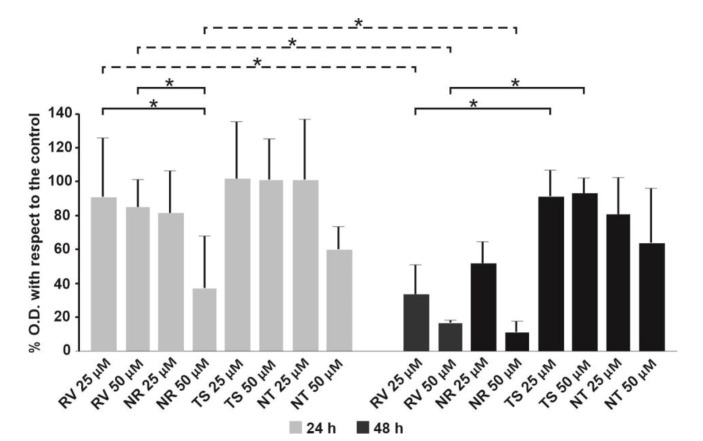
Toxicity towards T24 cells treated with RV and TS (alone or loaded in NEs at distinct concentrations) for 24 and 48 h measured by MTT assay. * *p* < 0.01.

Due to the increase in NE size, which, after loading with RV, enlarge from 30 nm to 140 nm, TS, which was similar in structure to RV, but without the 3 hydroxyl groups, was designed to load NEs with comparable sizes and used as a control. The cytotoxic effect of the compounds on the bladder cancer cell line was evaluated by the MTT assay. Whereas no significant difference was detectable at 24 h between free RV and TS with respect to untreated cell monolayers, on the contrary, a significant decrease in cell viability was revealed for the NEs loaded with RV compared to free RV at the concentration of 50 µM (Figure 7).

The treatment of monolayers for 48 h with the different formulations highlighted that the concentration of 25 µM of RV, both alone and loaded in NEs, was already able to induce a significant cell toxicity compared to the untreated control cells.

The results obtained, comparing 24 and 48 h incubation, showed that free RV induced a significant dose- and time-dependent increase in cytotoxicity, whereas RV-loaded NEs showed a faster activity, already inducing a high level of cytotoxicity at 24 h. These data were confirmed by Trypan Blue assay as compared to control cells, after treatment with the two RV concentrations (50 and 25 µM) at different time intervals (24 and 48 h) (data not shown).

Similarly to that observed by Bai et al. [9,32], the exposure of the T24 bladder cells to NR was able to induce cytotoxicity both at 24 and 48 h, whereas free RV seemed to influence cell viability only at 48 h. When 50 μM RV was loaded in NEs, a significant cell death was already induced at 24 h treatment, raising higher levels at 48 h treatment. From these results, it is possible to hypothesize a faster cellular uptake of RV-loaded NEs compared to free RV. Cell toxicity seems to be due to a different time of RV internalization that leads to higher intracellular drug concentration in a shorter time when loaded in NEs. 

It has been demonstrated that treatment of bladder cancer cells with high doses of RV (>25 µM) resulted in a significant decrease in cell viability by inducing apoptosis and cell cycle arrest [9,33,34]. On the other hand, at low concentrations (<20 µM), RV is able to inhibit apoptotic cell death, providing, thereby, protection against various diseases, including myocardial ischemic reperfusion injury, atherosclerosis, and ventricular arrhythmias [35]. Furthermore, studies performed by Stocco et al. [34] described a biphasic effect of RV on the cell viability of ECV304 cells, a derivation from the human bladder carcinoma T24 cell line, since, at high concentrations (>20 µM), it induced death cell, whereas at low concentrations (0.1–20 µM), it did not and also protected them from oxidative stress. According to these studies, it could be supposed that the T24 cell death we observed is linked to intracellular RV concentration if loaded and then released by NEs.

### 3.7. Evaluation of the Oxidative Stress of T24 Cells Treated with Free RV or Loaded in NEs

**Figure 8:**
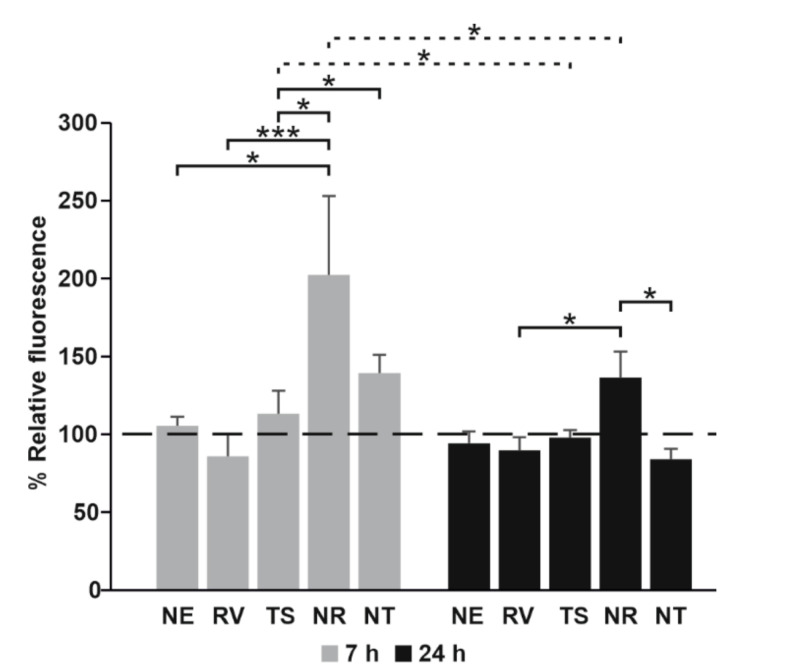
Color coded bar plots showing the oxidative stress of T24 cells exposed to RV and TS alone or loaded in NEs. Values were reported as mean ± SD. The presence of statistically significant differences among conditions (continuous line) and for each condition, between consecutive time points (dotted line), was also reported. * *p* < 0.05, *** *p* < 0.0001.

The production of reactive oxygen species in T24 cells, treated with RV and TS, alone or loaded in NEs (50 μM), was verified by using the H_2_DCFDA dye at two different times. As can be seen from Figure 8, while free RV was not able to induce any change in ROS production, if loaded in NEs, it induced strong oxidative stress in T24 cells at 7 and 24 h.

The evaluation of ROS production appeared to confirm the role of RV intracellular concentration. RV is an excellent scavenger of hydroxyl, superoxide, and other radicals. It also protects against lipid peroxidation in cell membranes and DNA damage caused by the generation of reactive oxygen species [36]. However, RV is also able to increase intracellular ROS production, leading to cell death. In our cell system, the high level of oxidative stress observed after 7 h of treatment with RV NEs leads us to suppose that the intracellular concentration of RV loaded and then released by NEs was at least higher than 20 μM. This hypothesis was also supported by RV release studies showing that the maximum release was within 8 h. Decreased cell viability caused by RV could then be mediated by ROS production and subsequent mitochondrial depolarization, as also observed from Yang et al. (2014) for capsaicin activity on T24 cells [37].

## 4. Conclusions

The application of Resveratrol in the pharmaceutical field is a disadvantage due to its poor water solubility and low stability, with a consequent low bioavailability. In order to overcome this drawback, RV encapsulation in NEs represents a promising strategy. Due to the large surface area provided by the nanometric size of the oil droplets of O/W NEs, it is possible to obtain a high loading capacity and enhanced solubility, which leads to an increased bioavailability of hydrophobic drugs and bioactive molecules. Moreover, the high energy preparation methods of NE employed are simple, efficient and do not require any sophisticated instruments.

The antiproliferative and antitumor activities of RV have been described on cell lines from different origins and also on human T24 tumor cells. Furthermore, some authors demonstrated that RV have a selective action towards urothelial cancer cells [38] compared to normal urothelial cells. Our results showed that developed NEs could ensure the maintenance of the biological activity of RV and a high efficiency of RV delivery into cancer cells, suggesting their possible applicability in biological systems and clinical practices.

## Figures and Tables

**Table 1:** Sample compositions.

Sample	Neem Oil (mg/mL)	Tw 20 (mg/mL)	RV (mg/mL)	TS (mg/mL)
N	9.2	18.4	-	-
NR			2.5	-
NT			-	2.5

**Table 2:** Chemical physical features of all samples.

Sample	Hydrodynamic Diameter (nm) ± SD	ζ-Potential (mV) ± SD	PDI ± SD	RV (mg/mL)	TS (mg/mL)	Polarity (I1/I3)	Microviscosity (IE/I3)
N	38.8 ± 0.7	−15.7 ± 0.4	0.21 ± 0.1	-	-	1.19	0.94
NR	137.8 ± 0.5	−23.0 ± 0.7	0.22 ± 0.1	1.3	-	1.64	1.15
NT	156.2 ± 3.2	−32.6 ± 1.0	0.26 ± 0.1	-	1.1	1.70	1.03

## Supplementary Materials

The following are available online at https://www.mdpi.com/article/10.3390/nano11061569/s1. Table S1: Composition of all studied formulations in the ternary diagram; in bold, sample composition of the dark region, Figure S1: Transmission electron microscopy micrographs of microemulsion sample (panel A) and empty nanoemulsion droplets after sonication (Panel B), Figure S2: Stability studies of NT in terms of variation of hydrodynamic diameter and ζ-potential up to 30 days at two different storage temperatures, Figure S3: Biological stability studies of NR (Panel A) and NT (Panel B) in terms of variation of hydrodynamic diameter and ζ-potential up to 48 h at the temperature of 37 °C.
