# Supplementary material for: Resveratrol-Loaded Nanoemulsions: In Vitro Activity on Human T24 Bladder Cancer Cells
Source: Nanomaterials (Basel). 2021 Jun 15;11(6):1569. doi: 10.3390/nano11061569 (PMC8232283; doi:10.3390/nano11061569)
Supplement: Supplementary file 1 [file nanomaterials-11-01569-s001.zip › supplementary-1239582 -revised.pdf]

nanomaterials

Article

Resveratrol-Loaded Nanoemulsions: In Vitro Activity on Human T24 Bladder Cancer Cells

Federica Rinaldi [1,#], Linda Maurizi [2,#], Jacopo Forte [1], Massimiliano Marazzato [2], Patrizia Nadia Hanieh [1], Antonietta Lucia Conte [2], Maria Grazia Ammendolia [3,*], Carlotta Marianecci [1,*], Maria Carafa [1,#] and Catia Longhi [2,#]

[1] Dipartimento di Chimica e Tecnologie del Farmaco, Sapienza Università di Roma, P.le A. Moro 5, 00185 Roma, Italy; federica.rinaldi@uniroma1.it (F.R.); jacopo.forte@uniroma1.it (J.F.); patrizianadia.hanieh@uniroma1.it (P.N.H.); maria.carafa@uniroma1.it (M.C.)
[2] Dipartimento di Sanità pubblica e Malattie infettive, Sapienza Università di Roma, P.le A. Moro 5, 00185 Roma, Italy; linda.maurizi@uniroma1.it (L.M.); massimiliano.marazzato@uniroma1.it (M.M.); antoniettalucia.conte@uniroma1.it (A.L.C.); catia.longhi@uniroma1.it (C.L.)
[3] Centro Nazionale Tecnologie Innovative in Sanità Pubblica, Istituto Superiore di Sanità, viale Regina Elena, 299, 00162 Rome, Italy; maria.ammendolia@iss.it
* Correspondence: maria.ammendolia@iss.it (M.G.A.); carlotta.marianecci@uniroma1.it (C.M.)
[#] These authors equally contributed to the work.

Citation: Rinaldi, F.; Maurizi, L.; Forte, J.; Marazzato, M.; Hanieh, P.N.; Conte, A.L.; Ammendolia, M.G.; Marianecci, C.; Carafa, M.; Longhi, C. Resveratrol-Loaded Nanoemulsions: In Vitro Activity on Human T24 Bladder Cancer Cells. *Nanomaterials* **2021**, *11*, 1569. https://doi.org/10.3390/nano11061569

Academic Editor(s): Eleonore Fröhlich; Luciana Dini; Cristian Vergallo

Received: 13 May 2021
Accepted: 10 June 2021
Published: 15 June 2021

Publisher's Note: MDPI stays neutral with regard to jurisdictional claims in published maps and institutional affiliations.

Copyright: © 2021 by the authors. Submitted for possible open access publication under the terms and conditions of the Creative Commons Attribution (CC BY) license (http://creativecommons.org/licenses/by/4.0/).

Table 1. Composition of all studied formulations in the ternary diagram; in bold, sample composition of the dark region.

Neem (g)	Tween 20 (g)	Hepes Buffer (g)	Neem (%w/w)	Tween 20 (%w/w)	Hepes Buffer (%w/w)
**1**	**1**	**98**	**1**	**1**	**98**
**2**	**2**	**96**	**2**	**2**	**96**
**5**	**10**	**85**	**5**	**10**	**85**
**5**	**20**	**75**	**5**	**20**	**75**
**5**	**30**	**65**	**5**	**30**	**65**
**5**	**40**	**55**	**5**	**40**	**55**
**5**	**50**	**45**	**5**	**50**	**45**
**6**	**55**	**39**	**6**	**55**	**39**
**15**	**40**	**45**	**15**	**40**	**45**
**20**	**45**	**35**	**20**	**45**	**35**
**30**	**30**	**40**	**30**	**30**	**40**
**43**	**17**	**40**	**43**	**17**	**40**
**20**	**25**	**55**	**20**	**25**	**55**
**15**	**15**	**70**	**15**	**15**	**70**
**10**	**10**	**80**	**10**	**10**	**80**
**30**	**15**	**55**	**30**	**15**	**55**
**30**	**5**	**65**	**30**	**5**	**65**
**43**	**17**	**40**	**43**	**17**	**40**
**45**	**5**	**50**	**45**	**5**	**50**
**55**	**5**	**40**	**55**	**5**	**40**
**70**	**5**	**25**	**70**	**5**	**25**
**65**	**15**	**20**	**65**	**15**	**20**
**55**	**12**	**33**	**55**	**12**	**33**
**43**	**17**	**40**	**43**	**17**	**40**
**63**	**5**	**32**	**63**	**5**	**32**
**62**	**12**	**26**	**62**	**12**	**26**
**43**	**12**	**45**	**43**	**12**	**45**
**27**	**25**	**48**	**27**	**25**	**48**
**23**	**35**	**42**	**23**	**35**	**42**
75	5	20	75	5	20
70	12	18	70	12	18
55	30	15	55	30	15
40	40	20	40	40	20
35	38	27	35	38	27
30	48	22	30	48	22
20	58	22	20	58	22
10	55	35	10	55	35
2	68	30	2	68	30
10	50	40	10	50	40
38	22	40	38	22	40
30	40	30	30	40	30
32	32	36	32	32	36
45	30	25	45	30	25
45	25	30	45	25	30
55	20	25	55	20	25
65	15	20	65	15	20
70	20	10	70	20	10

20	52	28	20	52	28
8	62	30	8	62	30
25	55	20	25	55	20
85	5	10	85	5	10
75	15	10	75	15	10
65	25	10	65	25	10
55	35	10	55	35	10
55	40	5	55	40	5
45	50	5	45	50	5
20	70	10	20	70	10
10	65	25	10	65	25
5	75	20	5	75	20
30	60	10	30	60	10
35	50	15	35	50	15
15	80	5	15	80	5
5	90	5	5	90	5
32	41	26	32	41	26
14	62	24	14	62	24
47	40	13	47	40	13
90	5	5	90	5	5
5	80	15	5	80	15
2	96	2	2	96	2

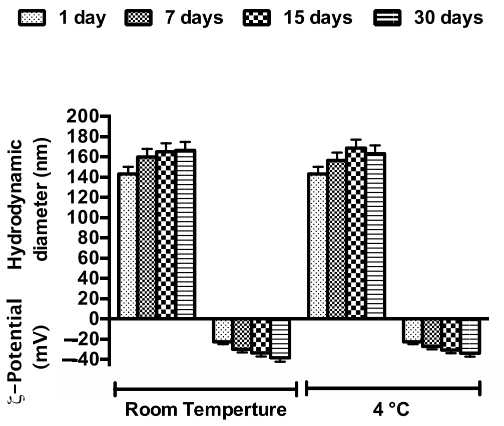

Figure S1. Stability studies of NT in terms of variation of hydrodynamic diameter and ζ-potential up to 30 days at two different storage temperatures.

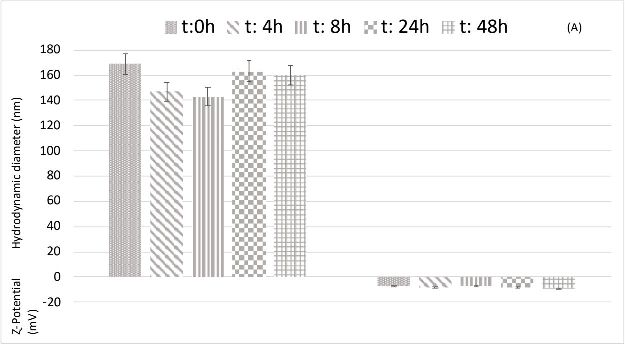

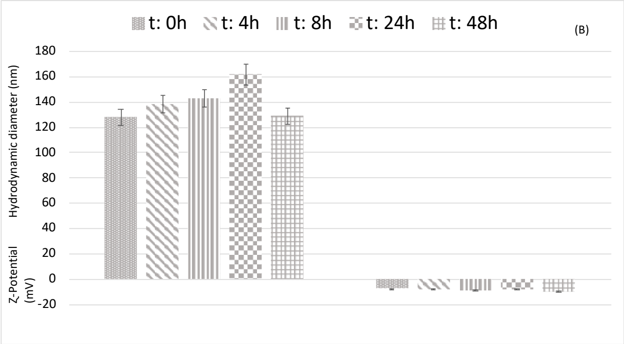

Figure S2. Biological stability studies of NR (**A**) and NT (**B**) in terms of variation of hydrodynamic diameter and ζ-potential up to 48 h at the temperature of 37 °C. .
